# Commentary: Physical therapy for sleep apnea: a smartphone application for home-based physical therapy for patients with obstructive sleep apnea

**DOI:** 10.3389/fneur.2024.1373229

**Published:** 2024-03-04

**Authors:** Carlos O'Connor Reina, Peter Baptista, Guillermo Plaza

**Affiliations:** ^1^Otorhinolaryngology Department, Hospital Quironsalud Marbella, Marbella, Spain; ^2^Otorhinolaryngology Department, Clínica Universitaria de Navarra, Pamplona, Spain; ^3^Otorhinolaryngology Department, Hospital Universitario de Fuenlabrada, Universidad Rey Juan Carlos, Madrid, Spain; ^4^Otorhinolaryngology Department, Hospital Sanitas La Zarzuela, Madrid, Spain

**Keywords:** obstructive sleep apnea, home-based physical therapy, smartphone application, physical therapy, respiratory muscle training

## Background

Obstructive sleep apnea (OSA) is the most common respiratory disease, with an increasing incidence worldwide. Telemedicine based on smartphone apps to treat this disease seems worthwhile. Myofunctional therapy is one of the options to treat OSA, and it has been recommended only for specific cases seeking alternative treatments and who are reluctant to undertake surgical or mechanical strategies (1).

## State of the art

**Figure 1:**
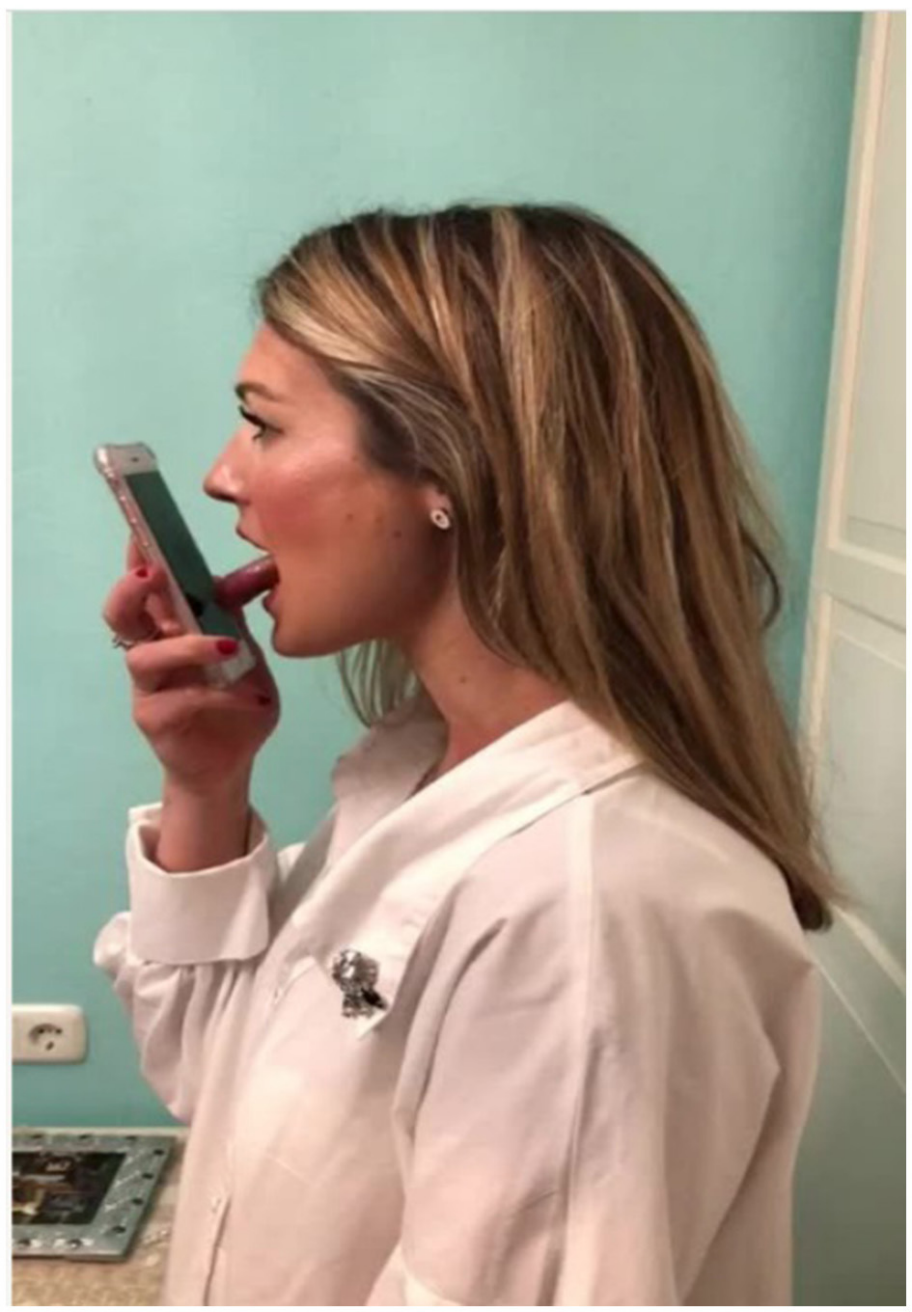
Video of a real person performing an oropharyngeal exercise.

In this journal, a recent manuscript published by Bui-Diem et al. (2) raised some issues we would like to address here. Our group designed an app called Airway Gym (3) to treat obstructive sleep apnea (OSA) that promotes proprioceptive rehabilitation and coordination of the airway muscles (4). This app includes nine exercises based on myofunctional therapy aimed at improving the tonicity of the various muscles involved in the pathogenesis of OSA (5). Before each exercise, an animated demonstration and a video with a real person are shown to the patient (Figure 1) so that they learn how to perform the exercise. After each exercise, the patient receives visual, acoustic, and tactile feedback about the success of their performance as a point score. When the patient finishes the exercises, the results are saved on a networked online storage (in the cloud), and a therapist can evaluate the patient's adherence and performance of the exercises. Users of the app can follow the progress of their daily activity over time. A chat function is available through which the patient can contact the therapist directly. Additional information can be found on the AirwayGym webpage https://airwaygym.app/en/gymnasts-homepage.

## Randomized clinical trial with an app

We have already performed a randomized clinical trial (6) where the intervention group with severe OSA showed significant improvements in most metrics of OSA scores. The control group just performed sham therapy. The apnea-hypopnea index decreased by 53.4% from 44.7 (range 33.8–55.6) to 20.9 (14.0–27.7) events/h (*p*-value < 0.001). The oxygen desaturation index decreased by 46.5% from 36.3 (27.2–43.4) to 19.4 (12.9–26.0) events/h (*p* = 0.003). The Epworth Sleepiness Scale score decreased from 10.3 (8.7–12.2) to 5.4 (3.4–7.3) in the app group (*p* < 0.001). Since Eckert (7) defined non-anatomical factors or 'phenotypes' as crucial determinants of OSA for many people, our group has focused on investigating those with a weakness in pharyngeal dilator muscle control known as “Hypotonic.” Studies performed with our app demonstrated that this was the best phenotype to improve adherence and receive myofunctional therapy (8).

In their article, Bui-Diem et al. (2) designed an app that, to the best of their knowledge, is the first application designed to assist patients with OSA in performing rehabilitation programs at home. However, they mentioned Airway Gym in their article and considered it an application for sleep apnea to practice upper airway muscle strength, although they erroneously mentioned that the video of our app does not show a real person. Furthermore, the use of their app (1) is clearly very similar to how we use ours, and they did not reference any of our works that would support this assertion.

## Conclusion

We believe that our app has been underestimated by the authors and truly was the first designed to perform a rehabilitation program at home using real-person videos based on enhanced tone and proprioceptive deficit of upper airway muscles in OSA patients. Future publications by Bui-Diem et al. should always reference articles on which they have based their idea; in this case, we consider it based on the concept from our research.

## Ethics statement

Written informed consent was obtained from the individual(s) for the publication of any identifiable images or data included in this article.

## Author contributions

CO'C: Writing – original draft, Writing – review & editing. PB: Conceptualization, Writing – original draft. GP: Investigation, Writing – review & editing.
